# BDNF promotes activation of astrocytes and microglia contributing to neuroinflammation and mechanical allodynia in cyclophosphamide-induced cystitis

**DOI:** 10.1186/s12974-020-1704-0

**Published:** 2020-01-13

**Authors:** Honglu Ding, Jialiang Chen, Minzhi Su, Zhijun Lin, Hailun Zhan, Fei Yang, Wenbiao Li, Juncong Xie, Yong Huang, Xianguo Liu, Bolong Liu, Xiangfu Zhou

**Affiliations:** 1 Department of Urology, the Third Affiliated hospital of Sun Yat-Sen University, 600 Tianhe Rd, Guangzhou, 510630 China; 2 Department of Rehabilitation, The Third Affiliated Hospital and Lingnan Hospital of the Sun Yat-Sen University, 2693 Kaichuang Rd, Guangzhou, 510700 China; 3 Pain Research Center and Department of Physiology, Zhongshan School of Medicine of Sun Yat-sen University, 74 Zhongshan Rd. 2, Guangzhou, 510080 China; 4 Guangdong Provincial Key Laboratory of Brain Function and Disease, 74 Zhongshan Rd. 2, Guangzhou, 510080 China

**Keywords:** Cystitis, BDNF, TrkB, Neuroinflammation, Mechanical allodynia, Astrocytes, Microglia

## Abstract

**Background:**

Patients with interstitial cystitis/bladder pain syndrome (IC/BPS) often grieve over a low quality of life brought about by chronic pain. In our previous studies, we determined that neuroinflammation of the spinal dorsal horn (SDH) was associated with mechanisms of interstitial cystitis. Moreover, it has been shown that brain-derived neurotrophic factor (BDNF) participates in the regulation of neuroinflammation and pathological pain through BDNF-TrkB signaling; however, whether it plays a role in cyclophosphamide (CYP)-induced cystitis remains unclear. This study aimed to confirm whether BDNF-TrkB signaling modulates neuroinflammation and mechanical allodynia in CYP-induced cystitis and determine how it occurs.

**Methods:**

Systemic intraperitoneal injection of CYP was performed to establish a rat cystitis model. BDNF-TrkB signaling was modulated by intraperitoneal injection of the TrkB receptor antagonist, ANA-12, or intrathecal injection of exogenous BDNF. Mechanical allodynia in the suprapubic region was assessed using the von Frey filaments test. The expression of BDNF, TrkB, *p*-TrkB, Iba1, GFAP, *p*-p38, *p*-JNK, IL-1β, and TNF-α in the L6-S1 SDH was measured by Western blotting and immunofluorescence analysis.

**Results:**

BDNF-TrkB signaling was upregulated significantly in the SDH after CYP was injected. Similarly, the expressions of Iba1, GFAP, *p*-p38, *p*-JNK, IL-1β, and TNF-α in the SDH were all upregulated. Treatment with ANA-12 could attenuate mechanical allodynia, restrain activation of astrocytes and microglia and alleviate neuroinflammation. Besides, the intrathecal injection of exogenous BDNF further decreased the mechanical withdrawal threshold, promoted activation of astrocytes and microglia, and increased the release of TNF-α and IL-1β in the SDH of our CYP-induced cystitis model.

**Conclusions:**

In our CYP-induced cystitis model, BDNF promoted the activation of astrocytes and microglia to release TNF-α and IL-1β, aggravating neuroinflammation and leading to mechanical allodynia through BDNF-TrkB-p38/JNK signaling.

## Background

Interstitial cystitis/bladder pain syndrome (IC/BPS) is an enigmatic chronic inflammatory disease of the bladder. It is characterized by bladder pain accompanied with frequency, urgency, nocturia, and sterile urine, excluding typical urinary tract infections [1–3]. Chronic pain symptom affects the patients’ quality of life severely. A community-based study in the USA reported a high prevalence rate of BPS, 2.7–6.5% in women, and 2–4% in men [4, 5]. These rates typically vary between 6 and 32% in different countries [6]. Although a variety of theories have been proposed, including bladder urothelial defects or dysfunction, mast cell activation and autoimmunity, the etiology and pathophysiology remain largely unexplored [2, 7].

Our previous study demonstrated that neuroinflammation in the spinal dorsal horn (SDH) might be associated to mechanism of interstitial cystitis, and contribute to mechanical allodynia [8, 9]. Neuroinflammation is a complex and well-coordinated process consisting of various glial cells in the central nervous system (CNS) and peripheral immune cells [10]. Astrocytes and microglia are two important glial cells that participate actively during the neuroinflammatory process by releasing proinflammatory cytokines such as interleukin-1β (IL-1β), interleukin-6 (IL-6), and tumor necrosis factor-α (TNF-α), etc. [11, 12]. Mitogen-activated protein kinases (MAPK) is a family of serine/threonine kinases that include p38 and Jun N-terminal kinase (JNK) signaling pathways; they are activated and phosphorylated in astrocytes and microglia, participating in the release of proinflammatory cytokines [13, 14]. A large amount of evidence suggests that neuroinflammation can lead to and aggravate pathological pain [15]. Previous studies have reported that BDNF-TrkB signaling can affect pain modulation by regulating neuroinflammation in some pathological pain models [16, 17].

Brain-derived neurotrophic factor (BDNF) is a major neurotrophic factor in the CNS, and plays an important role in learning and memory formation [18]. Also, BDNF plays a crucial role in the occurrence and development of neuroinflammation [11]. BDNF is regulated by neuronal activity and stored in dense-core synaptic vesicles at the terminals of the neurons [19]. After a nerve injury or inflammation in the spinal cord, dorsal root ganglia, or other related areas, BDNF is overexpressed and released from neurons [20]. The increased release of BDNF contributes to synaptic plasticity and central sensitization, thereby participating in the development of chronic pain [21, 22]. Tyrosine-protein kinase B (TrkB) is a high-affinity BDNF receptor [19], which is reportedly expressed in neurons, microglia, and astrocytes [23]. BDNF mediates its action through various signaling pathways triggered by the activation of the TrkB receptor.

However, whether and how BDNF-TrkB signaling in the SDH regulates neuroinflammation and mechanical allodynia in cystitis remains unknown. In this investigation, we present novel evidence showing how astrocytes and microglia activated to increase neuroinflammation and mechanical allodynia in our CYP-induced cystitis model through BDNF-TrkB-p38/JNK signaling.

## Methods

### Animals

All experiments were approved by the Institutional Animal Care and Use Committee at the Sun Yat-Sen University and performed according to the National Institutes of Health Guide for the Care and Use of Laboratory Animals (National Institutes of Health publication no. 85–23; revised 1985). Adult female Sprague-Dawley rats with an approximate weight of 200–220 g were purchased from the Institute of Experimental Animals at the Sun Yat-Sen University. All rats were randomly assigned to each group and housed in the separate licensed animal units at a temperature of 24 °C and 12 h day/night cycle. Food and water were always available and libitum.

### Animal model of cystitis

CYP (25 mg/kg; Sigma) was systemic injected intraperitoneally (i.p.) on the first, fourth, and seventh day, to establish the chronic cystitis rat model [9]. On the eighth day after the first CYP injection, a urodynamic test and a hematoxylin-eosin staining assay of the bladder tissue was conducted to verify the successful establishment of the model.

### Chemicals

ANA-12 (0.5 mg/kg; HY-12497, MedChem Express, USA), injected intraperitoneally(i.p.), was dissolved in 10% dimethyl sulfoxide (DMSO) [24, 25]. K252a (2 μg/10 μL/rat; #12754, Cell Signaling Technology), injected intrathecally (i.t.), was dissolved in 10% DMSO [26]. Recombinant human BDNF protein (3 ng/10 μL/rat; 248-BD-025, R&D systems, Minneapolis, MN), injected intrathecally (i.t.), was reconstituted at 25 μg/mL in 0.1 M sterile phosphate-buffered saline (PBS, pH 7.4) [27, 28].

### Intrathecal injection

Intrathecal injection was conducted as previously described [9, 29]. Briefly, rats were lightly anesthetized using 3% iIsoflurane, and a 25-gauge needle was inserted into the intervertebral space between L5 and L6 after the skin was sterilized with 75% alcohol. A flicking of the tail was considered as a successful puncture. The needle was left in the dosing position for over 15 s after administration to ensure that the drug was delivered.

### Experimental design

To verify our conjecture, we designed and employed the experiments described below.

Firstly, for the time gradient experiment, we divided rats into four groups: the control group, which was injected intraperitoneally with 0.9% normal saline, and the remaining groups, which were injected with CYP as previously described to establish the cystitis model. Of the non-control groups, one was sampled on the eighth day, the second was sampled on the 12th day, and the third on the 18th day, respectively. In order to ensure the same sampling time, we controlled when the first CYP injection took place.

Secondly, for the antagonist experiment, we divided it into three parts. In part 1, we set the concentration gradient of ANA-12 including ANA-12 (0.1 mg/kg), ANA-12 (0.5 mg/kg), and ANA-12 (1 mg/kg). In part 2, we compared the treatment effect between ANA-12 (0.5 mg/kg) and K252a; they were administered every other day after CYP injection to ensure that normal modeling was not interfered with. In part 3, we administered ANA-12 (0.5 mg/kg) at three different time periods to study its treatment effects including the CYP + ANA-12 group (injected every other day after CYP injection), the CYP + ANA-12 group (injected next day following the third CYP injection, administrated for 3 days continuously), and the ANA-12 + CYP group (injected 1 day prior to CYP injection). Besides, we set the control group, the CYP group, and the CYP + 10%DMSO group in those experiments above.

Thirdly, for the exogenous BDNF experiment, we divided rats into four groups: the control group, the CYP group, the CYP + sterile PBS (10 μL i.t.) group, and the CYP + rBDNF group (injected every other day after CYP injection).

All experiments were repeated four times, with five rats in each group to ensure adequate tissue materials for Western blotting and immunofluorescence analysis and to obtain statistical significance. The rats from the antagonist experiment and the exogenous BDNF experiment were dissected under deep anesthesia to harvest the L6–S1 spinal cord on the twelfth day after the first CYP injection.

### Von Frey filaments test

Pelvic pain response was assessed through the von Frey filaments test. We performed mechanical withdrawal threshold assessments on the suprapubic region, which is reportedly an effective method for assessing hyperalgesia and mechanical allodynia in rat models with bladder hypersensitivity [9, 30, 31]. Measurements were taken using the up-down method with a series of von Frey filaments (0.6, 1, 1.4, 2, 4, 6, 8, and 15 g) [9]. Licking or scratching the area of stimulation, arising, or jumping up quickly were all regarded as a positive behavioral response. It is worth noting that with regards to the rats from the antagonist experiment and the exogenous BDNF experiment, when we conducted behavioral measurements and CYP injections on the same day, we performed the CYP injection after behavioral measurement.

### Western blotting

After the L6-S1 spinal cord was harvested, the SDH was separated and immediately stored at − 80 °C for further analysis. Samples were lysed in the fresh radio immunoprecipitation assay (RIPA) protein lysis buffer, containing a cocktail of proteinase and phosphatase inhibitors. Protein concentration was determined using the bicinchoninic acid (BCA) protein assay. Proteins were separated by sodium dodecyl sulfate-polyacrylamide gel electrophoresis (SDS-PAGE), for 30 min at 80 V and 60 min at 100 V, and subsequently transferred onto polyvinylidene fluoride (PVDF) membranes at 300 mA. After blocking with 5% skim milk for 1 h at 20–25 °C, the membranes were incubated overnight at 4 °C with the following primary antibodies: BDNF (1:1000; NB100-98682, Novus Biological), TrkB (1:1000; #4603, Cell Signaling Technology), phospho-TrkB (Tyr515, 1:1000; AF3462, Affinity Biosciences), ionized calcium-binding adapter molecule 1 (Iba-1, 1:1000; ab5076, Abcam), glial fibrillary acidic protein (GFAP, 1:1000; #3670, Cell Signaling Technology), phosphor-p38 (Tyr180/182, 1:1000; #4511, Cell Signaling Technology), phosphor-JNK (Thr183/Tyr185, 1:1000; #9251s, Cell Signaling Technology), IL-1β (1:2000; ab9722, Abcam), TNF-α (1:1000; ab66579, Abcam) and β-actin (1:1000; #3700, Cell Signaling Technology). After three washes with TBST, the membranes were incubated with the secondary antibodies conjugated to horseradish peroxidase for 1 h at 20–25 °C. Protein bands were detected with an enhanced chemiluminescence (ECL) kit. The band density was quantified via a computer-assisted imaging analysis system (ImageJ). Additionally, when necessary, we used Restore™ PLUS Western Blot Stripping Buffer to differentiate between protein bands of similar molecular weight.

### Immunofluorescence

After perfused through the heart with PBS or 0.9% normal saline followed by 4% paraformaldehyde under sodium pentobarbital anesthesia (50 mg/kg, i.p.), the excised spinal cord was fixed in 4% paraformaldehyde for at least 30 min. Subsequently, the spinal cord segments were transferred into 30% sucrose to be fully dehydrated at 4 °C. The tissue was cut into 20-μm-thick sections. After three washes in PBS, sections were blocked with immunofluorescent blocking agent (Beyotime) for 1 h at 20–25 °C and then incubated with the following primary antibodies: BDNF (1:500; ab213323, Abcam), phospho-TrkB (Tyr816, 1:200; NBP1-03499, Novus Biological), OX-42 (1:500; ab1211, Abcam), NeuN (1:200; MAB377, Millipore), and GFAP(1:400; #3670, Cell Signaling Technology) at 4 °C overnight. The antibodies used above were all within their validity period. After three washes with PBS, the sections were incubated with the secondary antibodies conjugated with Cy3 or Alexa-488 without light for 1 h at 20–25 °C. Measuring and imaging were performed by using a Leica fluorescence microscope (Leica DFC350 FX camera). We used the same exposure, gain, and gamma every time to keep the images standardized. ImageJ was also used to quantify the fluorescent intensity of each image.

### Statistical analysis

SPSS 21.0 software was used for all statistical analyses. All data were expressed as the mean ± standard error of the mean (SEM). Data from the Western blot analysis and the immunofluorescence assay were analyzed via the Student’ *t* test, while a two-way analysis of variance (ANOVA) followed by the Sidak's multiple comparisons test was used to analyze the data obtained from the mechanical withdrawal threshold. A probability level of *p* ≤ 0.05 was considered statistically significant.

## Results

### BDNF-TrkB signaling was upregulated in our CYP-induced cystitis model

**Fig. 1:**
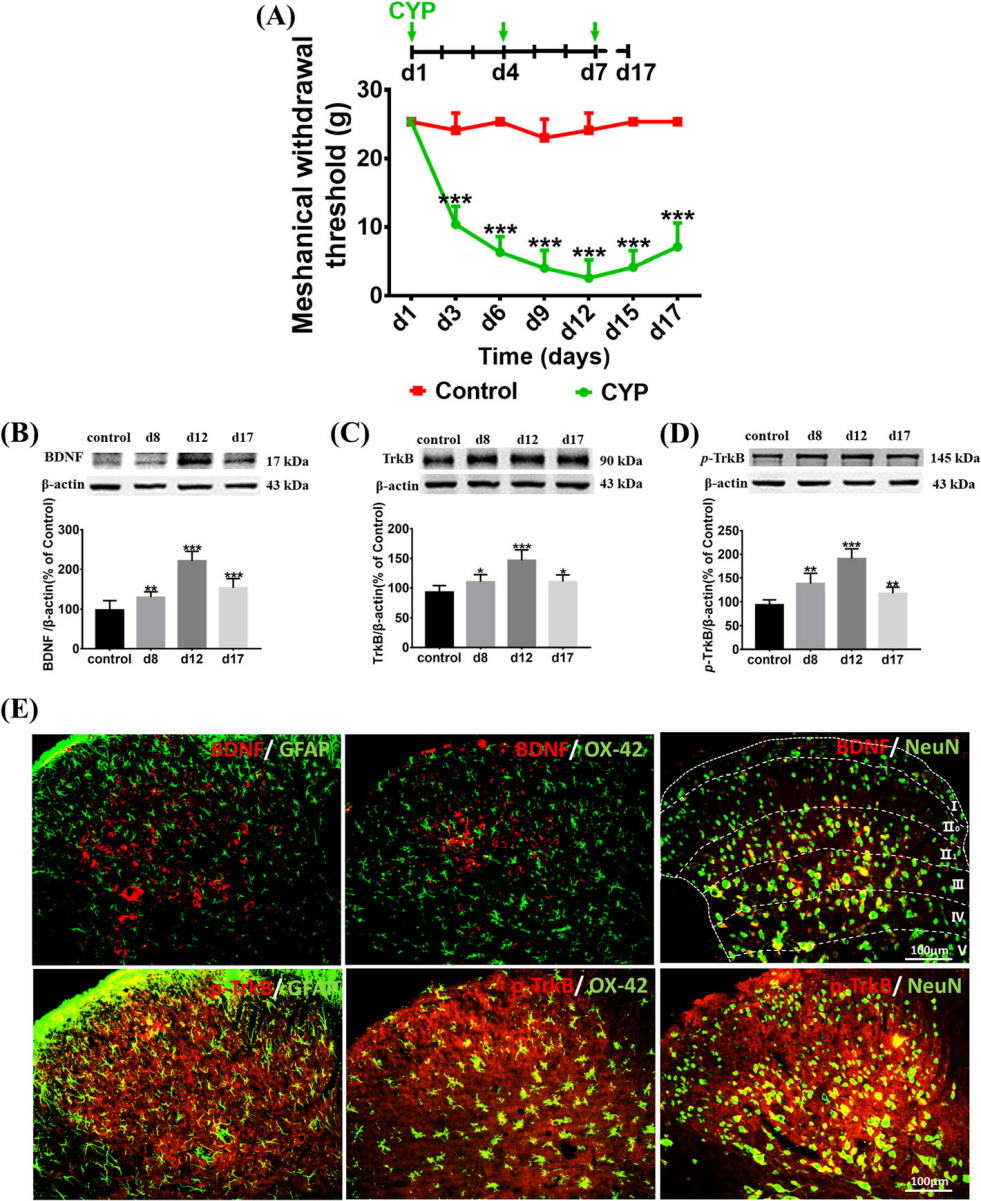
The expression of BDNF-TrkB signaling in the SDH of CYP-induced cystitis. **a** Changes of the mechanical threshold in CYP-induced cystitis model. Compared to that in the control group, the mechanical threshold of the cystitis group decreased significantly after the CYP injection and remained low until day 17, and the minimum threshold value was reached on day 12. The expression of **b** BDNF, **c** TrkB, and **d** *p*-TrkB were evaluated by western blots. Compared to the control group, they were upregulated on days 8, 12, and 17. e Immunofluorescence double staining assay of BDNF and *p*-TrkB in the SDH. BDNF and *p*-TrkB (red), NeuN, GFAP, and OX-42( green), co-localization (yellow). BDNF was mainly colocalized in neurons which mainly located in Laminate II to IV. And TrkB receptors expressed in neurons, microglia, and astrocytes. The white dotted lines in picture “BDNF/NeuN” showed the laminate of the SDH according to Rexed and Steiner. Scale bar = 100 μm. All data were calculated as mean ± SEM (*n* = 5 per group). *p < 0.05, ** *p* < 0.01, *** *p* < 0.001 vs. the control group

Through the von Frey filaments test, we were able to represent the pain curve. After comparing the results with the control group, the mechanical threshold of the cystitis group decreased significantly after the CYP injection and remained low until day 17. We also found that the minimum threshold value was reached on day 12 (Fig. 1a).

We conducted a Western blot analysis to measure the expression of BDNF-TrkB signaling in the SDH, and found that BDNF, TrkB, and *p*-TrkB were upregulated on days 8, 12, and 17 after the first CYP injection in the group with cystitis (Fig. 1b–d).

Furthermore, we conducted an immunofluorescence double staining assay to evaluate the localization of BDNF and p-TrkB in the SDH of the cystitis model. We found that BDNF was mainly colocalized with the neuronal marker NeuN but not with GFAP (the astrocytes marker) or OX-42(the microglia marker), and the co-stained neurons were mainly located in Laminate II to IV. Besides, we detected that p-TrkB was colocalized with NeuN, GFAP, and OX-42 (Fig. 1e).

### Astrocytes and microglia activated and promoted neuroinflammation through p38/JNK signaling in CYP-induced cystitis

**Fig. 2:**
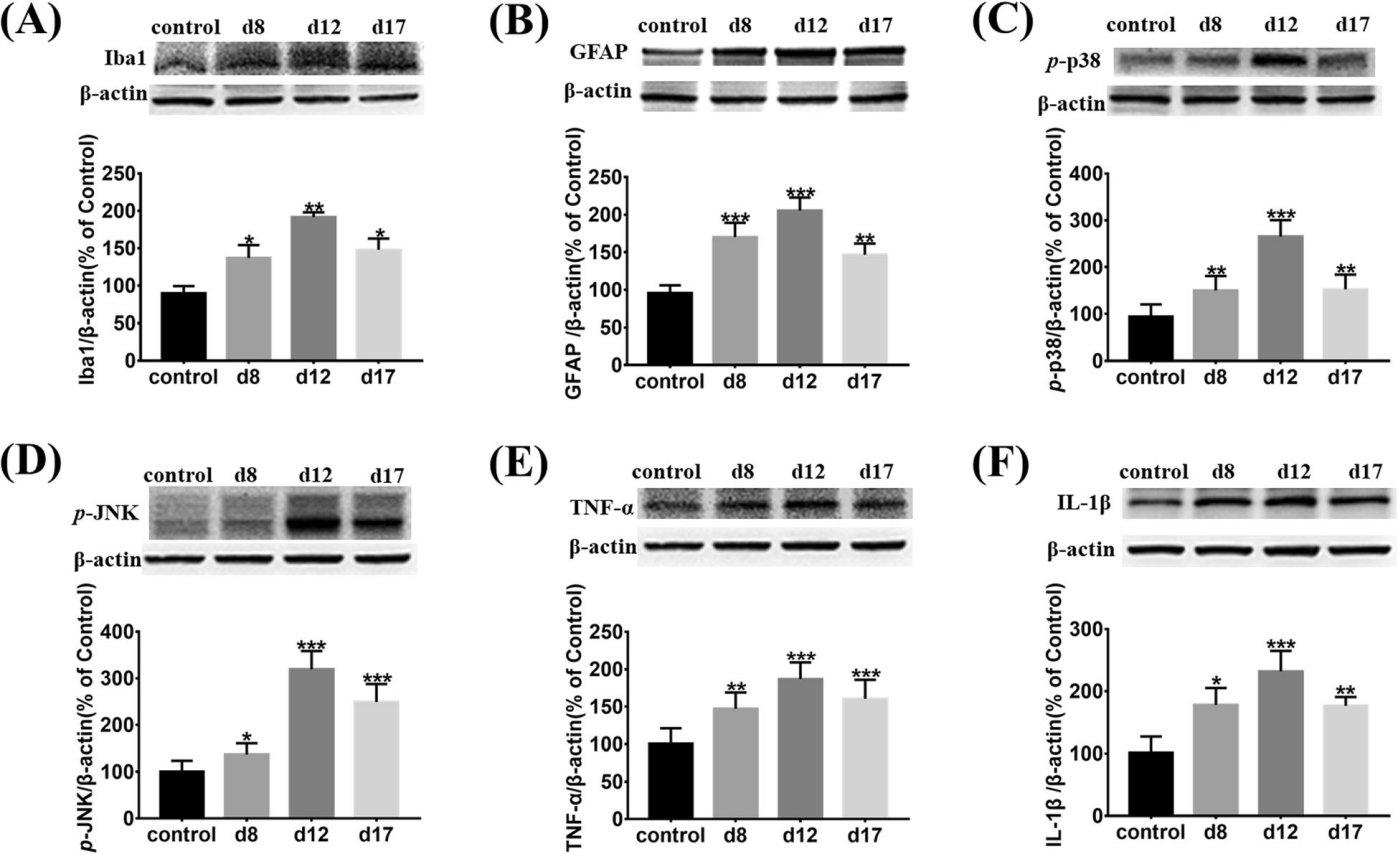
Astrocytes and microglia activated and released proinflammatory factors (IL-1β and TNF-α) through p38/JNK signaling in the SDH of CYP-induced cystitis. Western blots showing the expression of **a** Iba1, **b** GFAP, **c** *p*-p38, **d** *p*-JNK, **e** TNF-α, and **f** IL-1β. They were upregulated on days 8, 12, and 17 when compared with the control group. All data were calculated as mean ± SEM (*n* = 5 per group). *p < 0.05, **p < 0.01, ***p < 0.001 vs. the control group

From the Western blot analysis, we found that the expression of Iba1 and GFAP, which are the markers for microglia and astrocytes, respectively, were upregulated on days 8, 12, and 17 (Fig. 2a, b). By comparing immunofluorescence staining of the sample tissue, to that in that of the control group, OX-42 and GFAP staining were significantly increased in the SDH of cystitis group; moreover, activated microglia and astrocytes had hypertrophied cell bodies and significantly increased dendrites (Fig. 5).

IL-1β and TNF-α are two key proinflammatory cytokines that play roles in the induction and maintenance of neuropathic pain [11, 12]. We found that the expression of IL-1β and TNF-α were higher in the cystitis group than in the control group (Fig. 2e, f). The p38/JNK signaling pathway plays a key role in releasing proinflammatory cytokines by activation and phosphorylation of microglia and astrocytes [13, 14]. We also found that the expression of *p*-p38 and *p*-JNK of the cystitis group were upregulated when compared with the control group (Fig. 2c, d).

### Inhibition of BDNF-TrkB signaling attenuated mechanical allodynia in CYP-induced cystitis

**Fig. 3:**
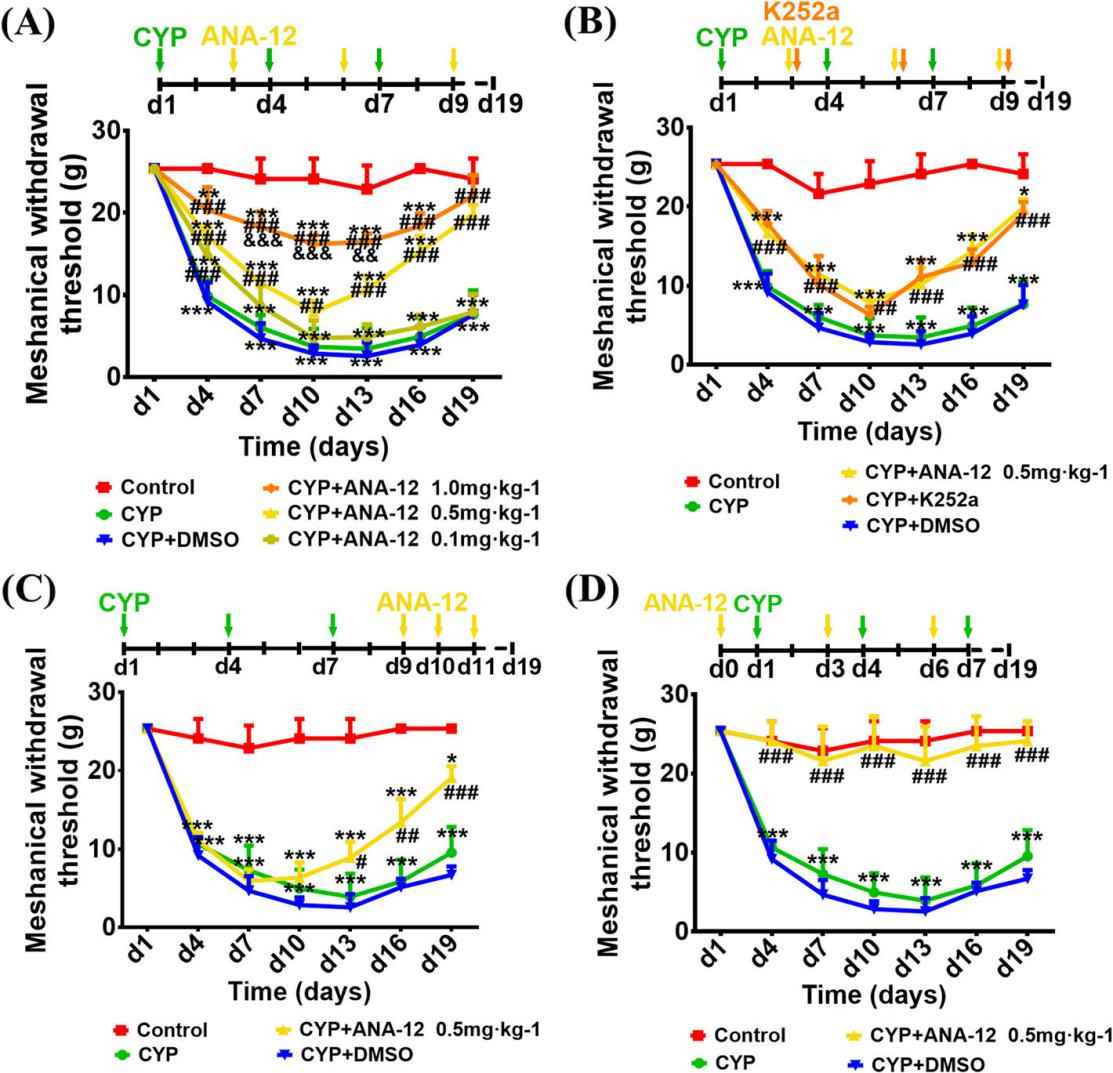
Changes in the mechanical threshold after antagonist administration. **a** Compared to the CYP + DMSO groups, ANA-12 (0.5 mg/kg), and ANA-12 (1.0 mg/kg) treated every other day after CYP injection reduced the decrease in the mechanical threshold and accelerated recovery significantly, while there was no difference between ANA-12 (0.1 mg/kg) and CYP + DMSO group. There was difference between ANA-12 (0.5 mg/kg) and ANA-12 (1.0 mg/kg) only at day 7, day 10, day 13. **b** Compared to the CYP+DMSO groups, ANA-12 (0.5 mg/kg), and K252a treated every other day after CYP injection reduced the decrease of the mechanical threshold and accelerated recovery significantly, and there was no difference between the effects of ANA-12 (0.5 mg/kg) and K252a. **c** ANA-12 treated next day after the third CYP injection 3 days continuously could reverse the mechanical threshold rapidly. **d** ANA-12 treated one day prior to CYP injection could prevent the onset of allodynia; moreover, there was no significant difference when compared with the control group. All data were analyzed using a two-way analysis of variance (ANOVA) followed by the Sidak's multiple comparisons test. All data were calculated as mean ± SEM (*n* = 10 per group). *p < 0.05, **p < 0.01, ***p < 0.001 vs. the control group. #*p* < 0.05, ##*p* < 0.01, ###*p* < 0.001 vs. the CYP + DMSO group. &&*p* < 0.01, &&&*p* < 0.001 vs. the ANA-12 (0.5mg/kg) group

Since we confirmed that CYP-induced cystitis overexpressed BDNF-TrkB signaling, we conducted some follow-up experiments to explore whether blocking the BDNF-TrkB signaling could alleviate the onset of allodynia in the cystitis model. As shown in Fig. 3a, when compared with the CYP + DMSO groups, ANA-12 (0.5 mg/kg) and ANA-12 (1.0 mg/kg) treated every other day after CYP injection reduced the decrease in the mechanical threshold and accelerated recovery significantly, while there was no difference between ANA-12 (0.1 mg/kg) and CYP + DMSO group. There was difference between ANA-12 (0.5 mg/kg) and ANA-12 (1.0 mg/kg) only at day 7, day 10, and day 13. As shown in Fig. 3b, when compared with the CYP + DMSO groups, K252a treated every other day after CYP injection reduced the decrease of the mechanical threshold and accelerated recovery significantly, and there was no difference between the effects of ANA-12 (0.5 mg/kg) and K252a. As shown in Fig. 3c, ANA-12 (0.5 mg/kg) treated the day after the third CYP injection over a continuous 3 days could reverse the mechanical threshold rapidly. Moreover, we also injected ANA-12 (0.5 mg/kg) 1 day prior to the CYP injection, and were surprised to find that it prevented the onset of allodynia (Fig. 3d).

### Inhibition of BDNF-TrkB signaling restrained activation of astrocytes and microglia and alleviated neuroinflammation in the SDH of CYP-induced cystitis

**Fig. 4:**
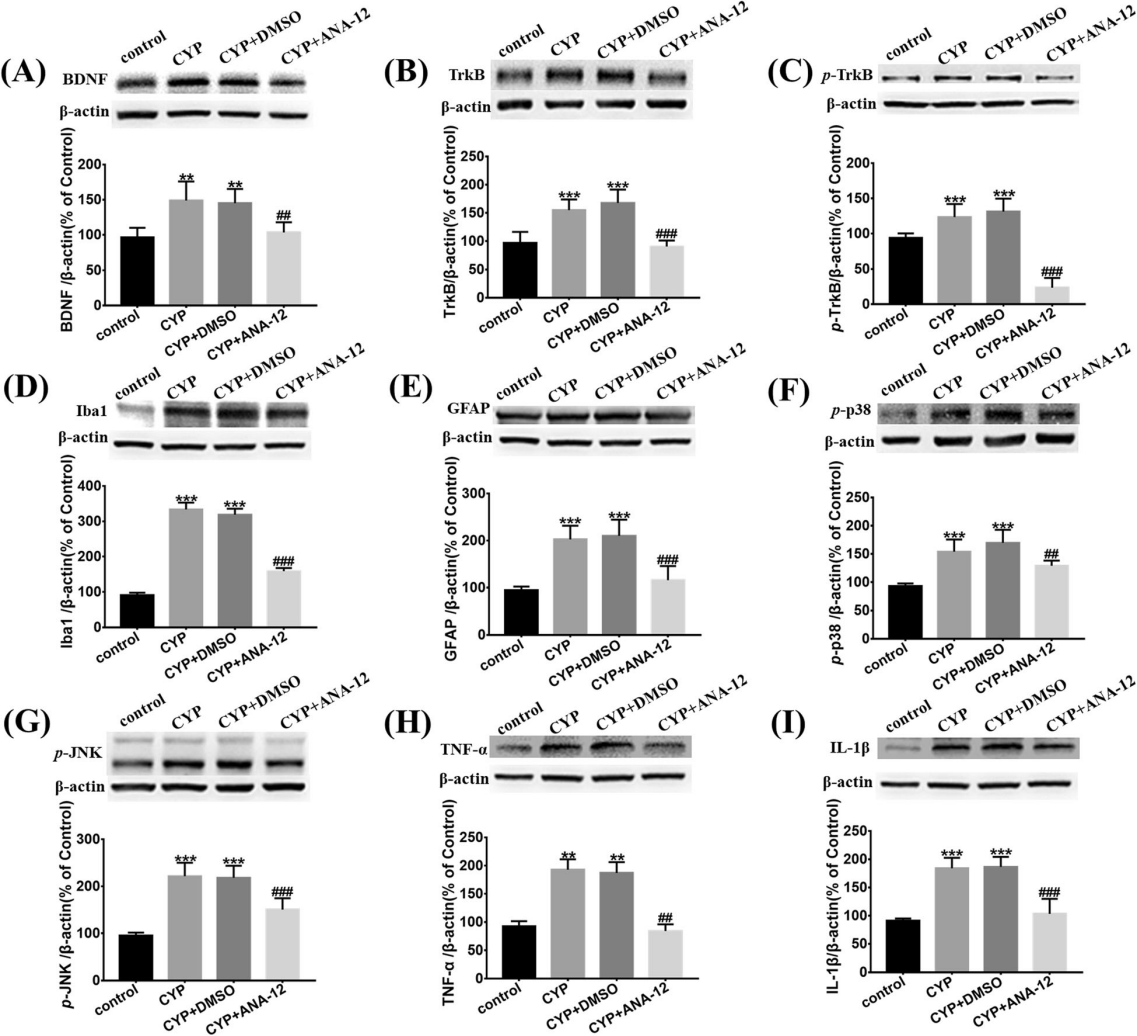
Antagonized TrkB could restrain the activation of astrocytes and microglia and supress the p38/JNK pathway to alleviate the release of IL-1β and TNF-α in the SDH of CYP-induced cystitis. Western blots showed that the overexpression of **a** BDNF, **b** TrkB, **c** *p*-TrkB, **d** Iba1, **e** GFAP, **f** *p*-p38, **g** *p*-JNK, **h** TNF-α, and **i** IL-1β were downregulated in comparison to the CYP + DMSO group after ANA-12 treatment. All data were calculated as mean ± SEM (*n* = 5 per group). **p < 0.01, ***p < 0.001 vs. control group. ##*p* < 0.01, ###*p* < 0.001 vs. CYP + ANA-12 group

For the Western blot analysis, we mainly used the L6-S1 SDH harvested on the 12th day after the first CYP injection, from the rats injected ANA-12 every other day. As is apparent in Fig 4a–c, the activation of TrkB was inhibited; surprisingly, we found that the expression of BDNF was downregulated as compared to the CYP + DMSO group.

**Fig. 5:**
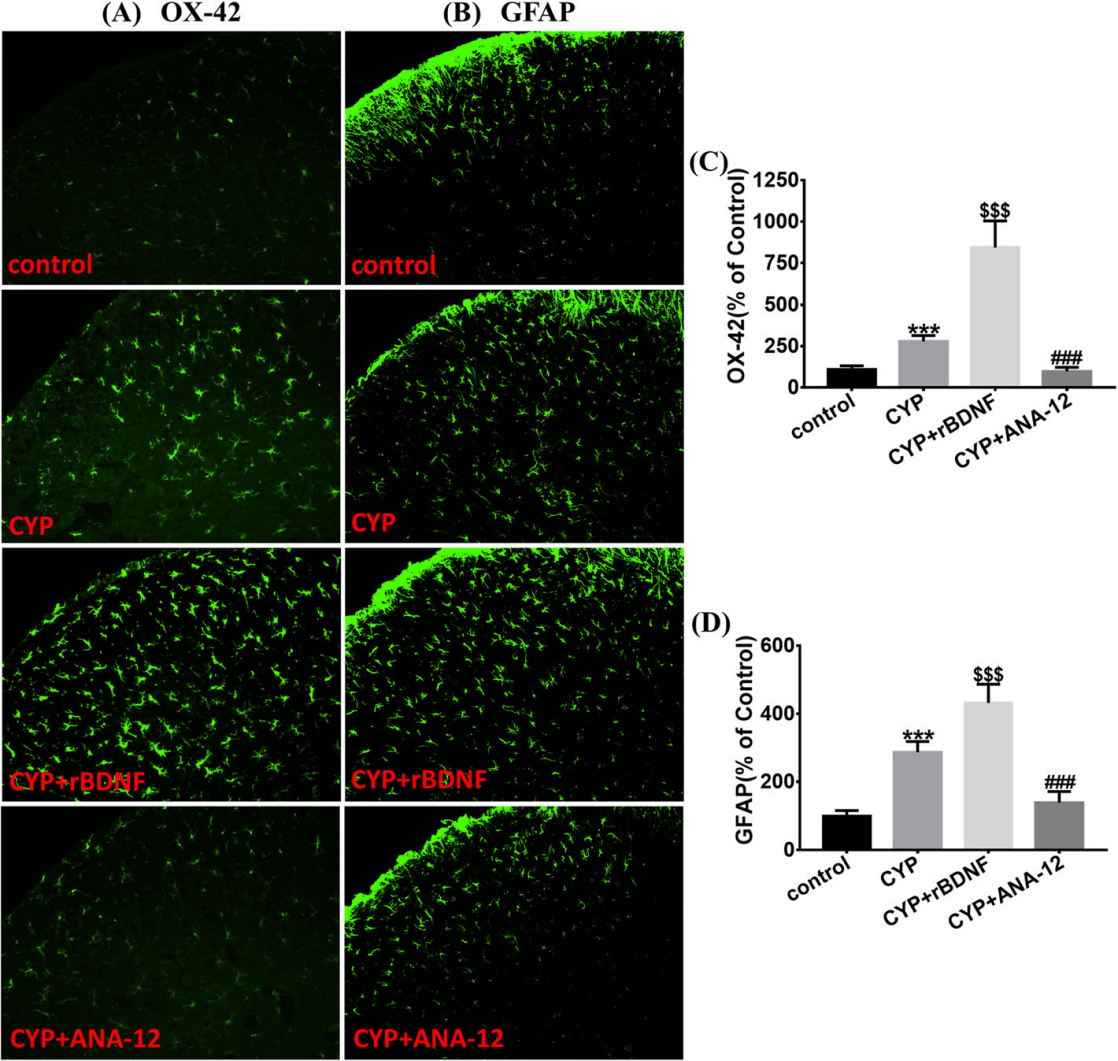
Immunofluorescence staining assay of astrocytes and microglia in the SDH. **a** Immunofluorescence staining of OX-42 (green), **b** Immunofluorescence staining of GFAP (green). Scale bar = 100 μm. When compared with the control group, OX-42 and GFAP staining were significantly increased in the SDH of CYP group; moreover, activated astrocytes and microglia presented hypertrophied cell bodies and significantly increased dendrites. **c**, **d** ANA-12 treatment restrained activation of astrocytes and microglia induced by CYP. The BDNF injection further promoted activation of astrocytes and microglia induced by CYP. All data were calculated as mean ± SEM (*n* = 5 per group). ***p < 0.001 vs. the control group, $$$*p* < 0.001 vs. the CYP + rBDNF group, ###*p* < 0.001 vs. the CYP + ANA-12 group

We also measured the expression and activation of astrocytes and microglia. As shown in Figs. 4d, e and 5, after ANA-12 treatment, the overexpression of Iba1 and GFAP were downregulated, and the activation of astrocytes and microglia were both inhibited significantly. From the Western blot analysis, after ANA-12 treatment, we determined that the overexpression of IL-1β, TNF-α, *p*-p38, and *p*-JNK were also suppressed (Fig. 4f–i).

### BDNF promoted activation of astrocytes and microglia and aggravated neuroinflammation and mechanical allodynia of CYP-induced cystitis

We administered exogenous BDNF by intrathecal injection for a more in-depth exploration of the role of BDNF in neuropathic inflammation and pathological pain of CYP-induced cystitis.

**Fig. 6:**
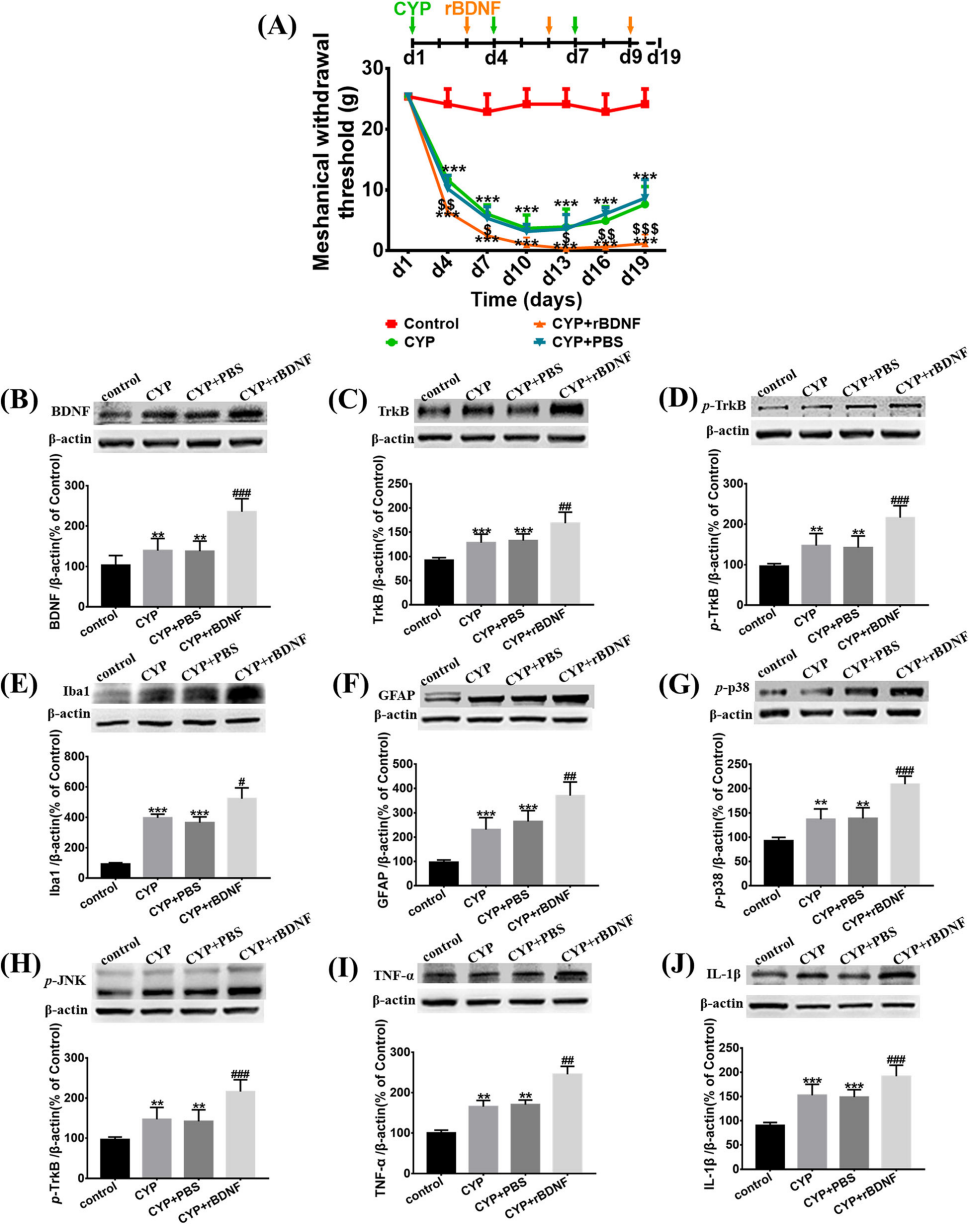
BDNF lowered the mechanical withdrawal threshold further and promoted activation of astrocytes and microglia, and enhanced the p38/JNK pathway to aggravate the release of IL-1β and TNF-α in the SDH of CYP-induced cystitis. **a** BDNF treated every other day after CYP injection could further lower the mechanical withdrawal threshold and suppress the retrieval of mechanical threshold when compared with the CYP + PBS group. After the exogenous BDNF injection, Western blots showing the expression of **b** BDNF, **c** TrkB, **d** *p*-TrkB, **e** Iba1, **f** GFAP, **g** *p*-p38, **h** *p*-JNK, **i** TNF-α, and **j** IL-1β were all further upregulated when compared with the CYP + PBS group. Data of mechanical withdrawal threshold were analyzed using a two-way analysis of variance (ANOVA) followed by the Sidak's multiple comparisons test. All data were calculated as mean ± SEM (*n* = 10 per group). **p < 0.01, ***p < 0.001 vs. the control group. #*p* < 0.05, ##*p* < 0.01, ###*p* < 0.001 vs. the CYP + rBDNF group. $*p* < 0.05, $$*p* < 0.01, $$$*p* < 0.001 vs. the CYP + PBS group

As demonstrated in Fig. 6a, treatment with BDNF could lower the mechanical withdrawal threshold further than what was seen in the CYP + PBS group. In addition, it could also suppress recovery of the mechanical threshold, at least within the time frame that we observed.

As determined by Western blot and immunofluorescence, the expression of TrkB and *p*-TrkB increased further, as compared to the CYP + PBS group (Fig. 6c, d). Astrocytes and microglia were activated further after the BDNF was administered (Figs. 5 and 6e, f). Meanwhile, the expression of IL-1β, TNF-α, *p*-p38, and *p*-JNK were further upregulated (Fig. 6g–j).

## Discussion

As an important urinary neurotrophic factor, BDNF is detected to be abnormally elevated in some lower urinary tract diseases, such as overactive bladder [32], stress urinary incontinence [33], and interstitial cystitis [34]. BDNF plays a specific role in urinary frequency and urgency without affecting capacity [35]. It has been reported that intrathecal injection of exogenous BDNF could shorten the urination interval and make urinary cycle disorder in a rat model; treatment with ANA-12, however, resulted in the opposite effect [36]. As nerve growth factor (NGF), BDNF is also a major neurotrophic factor in the CNS and plays a crucial role in the occurrence and development of neuroinflammation [11].

**Fig. 7:**
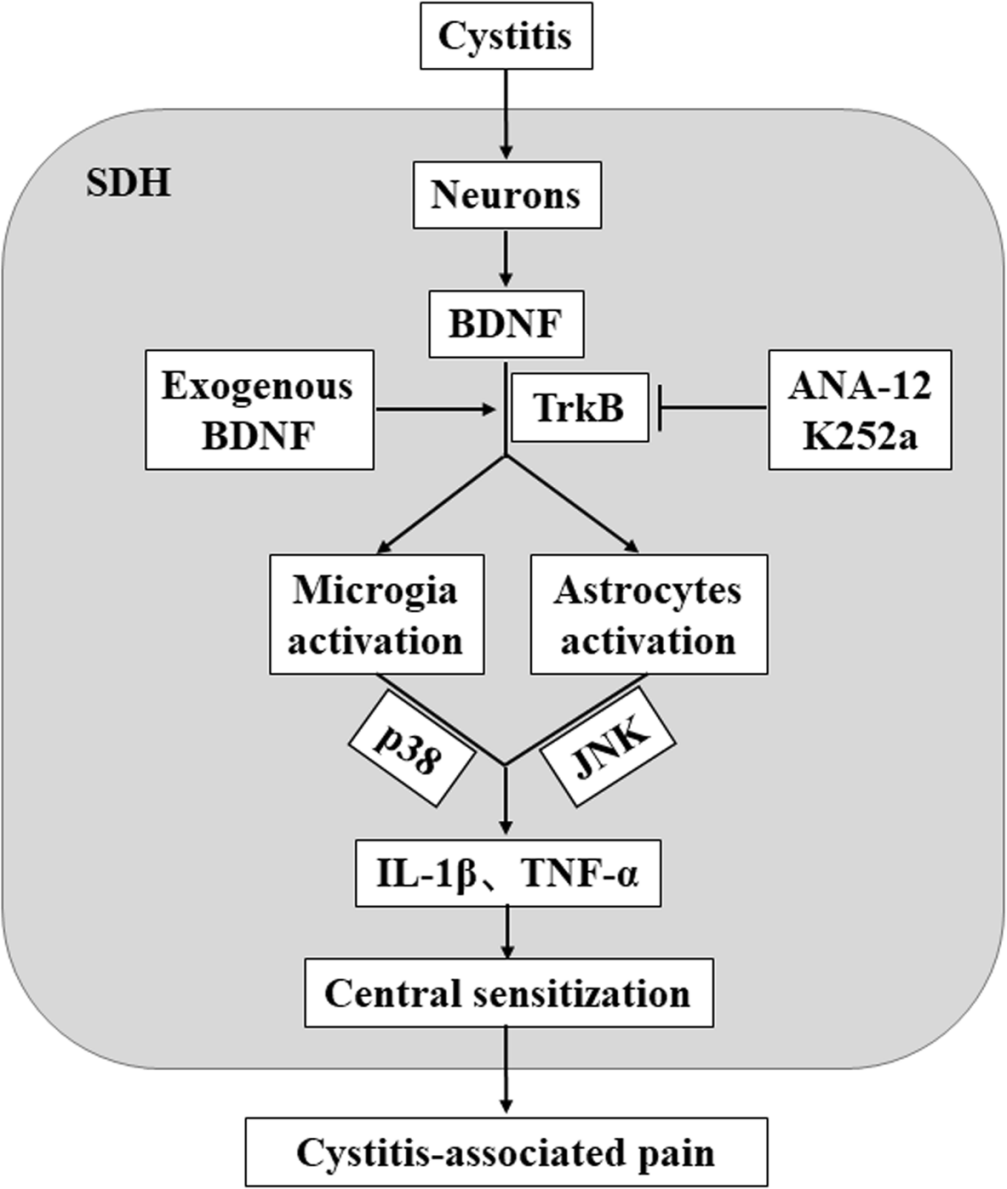
Schematic diagram of BDNF contributing to neuroinflammation and mechanical allodynia through the BDNF-TrkB-p38/JNK signaling pathway in cystitis

The conjecture that BDNF may promote activation of astrocytes and microglia to contribute to aggravating neuroinflammation and mechanical allodynia of CYP-induced cystitis through BDNF-TrkB signaling is the first time to be proposed. In the present study, we demonstrated that BDNF colocalized mainly in neurons, which coincides with precious reports that BDNF is regulated by neuronal activity and stored in dense-core synaptic vesicles at the terminals of these neurons [19]. The co-stained neurons were mainly located in Laminate II to IV according to Rexed [37] and Steiner [38], which indicated that BDNF is mainly synthesized and released by neurons located in Laminate II to IV after receiving the nociceptive signal triggered by CYP. Furthermore, we also detected that TrkB receptors expressed in neurons, microglia, and astrocytes, which was consistent with the results reported [23]. Meanwhile, the expression of BDNF, TrkB, *p*-TrkB, Iba1, GFAP, *p*-p38, *p*-JNK, IL-1β, and TNF-α in the L6-S1 SDH were all upregulated. ANA-12 is a potent and highly selective TrkB antagonist, which can cross the blood-brain-barrier and exert central TrkB blockade without compromising neuron survival [39]. Treatment with ANA-12 could reverse upregulation significantly and attenuate mechanical allodynia. Besides, intrathecal injection of exogenous BDNF could further promote activation of astrocytes and microglia, aggravate neuroinflammation, and mechanical allodynia. In summary, our study has validated the conjecture proposed previously; in our CYP-induced cystitis rat model, the neurons received noxious signals and released BDNF, which promoted activation of astrocytes and microglia to release proinflammatory factors through BDNF-TrkB-p38/JNK signaling pathway, thus, contributing to neuroinflammation and mechanical allodynia (Fig. 7).

In our study, we administered ANA-12 (0.5 mg/kg) at three different time periods to study its effects, and achieve good results. Further, 0.5 mg/kg of ANA-12 is a recognized concentration, which has been used in many studies [24, 25]. We tried three different concentrations of ANA-12; the result showed that 1.0 mg/kg of ANA-12 was more effective than the other two concentrations used and 0.1 mg/kg of ANA-12 had almost no therapeutic effect. Although the effect of ANA-12 (0.5 mg/kg) to reduce the decrease of the mechanical threshold was not as significant as ANA-12 (1.0 mg/kg), their final treatment effect is no different. Studies have shown that high doses of ANA-12 may be the induction of cell death [39]. Therefore, the therapeutic effect of ANA-12 (0.5 mg/kg) is more moderate. K252a is a TrkB receptor antagonist that has been used in many studies as well [26]; however, it can also inhibit the activation of phosphorylase kinase, protein kinase A (PKA), protein kinase C (PKC) [40], or even antagonize TrkA and TrkC receptors [41].

BDNF-TrkB signaling modulates the pain process by regulating neuroinflammation which has been reported in other pathological pain models as well [16, 17]. Glial cells play an important role in the development of neuroinflammatory and neuropathic pain. Astrocytes and microglia participate in the development of pathological neuroinflammatory processes by releasing proinflammatory cytokines (IL-1β, TNF-α) [11, 12]. Nonetheless, there is little evidence indicating whether there is a link between BDNF-TrkB signaling and activation of astrocytes and microglia. In our previous studies, we observed that the activation of astrocytes and microglia contribute to mechanical allodynia in CYP-induced cystitis model [8, 9]. As far as we know, Iba1 and GFAP are the main markers of microglia and astrocytes, respectively. Moreover, OX-42 and p38 are two indirect markers for the activation of microglia [42]. In the present study, the expression change of OX-42 and p-p38 were consistent with Iba1, both reflected an increase in activation of microglia. Astrocytes are the first glial cells identified as activated after peripheral nerve injury [43]. Astrocytes play an important role in the development and maintenance of some forms of chronic pain [44] and participate in chemotherapy-evoked behavioral changes [45]. Microglia plays a major role in chronic pain [46]. CYP-induced cystitis is considered a complete experimental model of cystitis [8, 9, 34]. Through systemic intraperitoneal injection of CYP 25 mg/kg every 3 days, we established a chronic cystitis model [9]. Antagonizing TrkB receptor could inhibit the activation of astrocytes and microglia significantly in the cystitis model. Besides, injection of exogenous BDNF could further promote the activation of microglia and astrocytes significantly. And the results of immunofluorescence double staining showed that TrkB receptors expressed in microglia and astrocytes. These results indicated that BDNF-TrkB signaling may be involved in the activation of astrocytes and microglia, which might be an important discovery for the study of astrocytes and microglia.

There are several signaling pathways reportedly implicated in the inflammatory responses regulating proinflammatory cytokines and chemokine production, including the nuclear factor kappaB (NF-κB) pathway, the MAPK pathway, and the activated protein 1 (AP-1) pathway [47]. MAPK is a family of serine/threonine kinases which include p38, JNK, and Erk [48]. Previous studies have reported that JNK [13] and p38 [14] are involved in the activation of both astrocytes and microglia. In our study, the expression of *p*-p38 and *p*-JNK were upregulated in the SDH of our CYP-induced cystitis model, suggesting the activation of p38/JNK signaling pathway. In addition, ASK1-dependent p38/JNK was reported as a downstream molecule of the BDNF signaling pathway [49]. BDNF-TrkB signaling regulates the progress of p38/JNK signaling. It is noteworthy to mention that BDNF intervenes with other signaling pathways, such as SP-NK1R signaling and Glu-NMDA signaling, which are two classic signaling pathways in neuroinflammation and pathological pain [50, 51]. Moreover, BDNF-TrkB signaling can affect the expression of GluN2B on neurons, which plays an important role in synaptic plasticity and hyperalgesia [52].

Interestingly, in the present study, we discovered that treatment with ANA-12 not only restrained the activation of TrkB but also downregulated the expression of BDNF, compared to the CYP + DMSO group. Previous research has found that, after activation, astrocytes and microglia can also produce and release BDNF except proinflammatory factors [53, 54]. It has also been reported that TNF-α can bind to TNFR1 on astrocytes and microglia to promote the release of BDNF [11, 55]. TNFR1 is also present on neurons, and binding with TNF-α can also stimulate the neuron to release BDNF [56]. Therefore, we hypothesized that with the release of TNF-α reduced or inhibited after the TrkB receptor antagonized, the “positive feedback” was restrained.

Nonetheless, there were some limitations to our study. When the ANA-12 treatment was applied, we mainly detected the changes occurring at the protein and tissue level in the L6-S1 SDH that was harvested from the rats treated every other day after CYP injection; however, we did not detect those changes in other two groups. In addition, we used OX-42 in the immunofluorescence assay to show microglial activity in the SDH since we could not find an available Iba1 antibody. Although OX-42 is an activated microglia marker, it may ignore changes in the ratio of the M1 and M2 phenotypes, and thus, fail to demonstrate complete activation of the microglia [57]. Further study is required to find a solution to these problems.

## Conclusion

Our results provide evidence that BDNF promotes activation of astrocytes and microglia to release TNF-α and IL-1β contributing to aggravating the neuroinflammation and mechanical allodynia through BDNF-TrkB-p38/JNK signaling in CYP-induced cystitis. Moreover, antagonizing TrkB receptors can alleviate neuroinflammation and mechanical allodynia significantly. These observations may provide a novel strategy for the study of astrocytes and microglia and treating cystitis.

## Data Availability

All data generated or analyzed during this study are included in this published article.

## Abbreviations

AP-1: Activated protein 1
BDNF: Brain-derived neurotrophic factor
BPS: Bladder pain syndrome
CNS: Central nervous system
CYP: Cyclophosphamide
DMSO: Dimethyl sulfoxide
GFAP: Glial fibrillary acidic protein
Iba-1: Ionized calcium-binding adapter molecule 1
IC: Interstitial cystitis
IL-1β: Interleukin-1β
JNK: Jun N-terminal kinase
MAPK: Mitogen-activated protein kinases
NF-Κb: Nuclear factor kappaB
NGF: Nerve growth factor
PBS: Phosphate-buffered saline
SDH: Spinal dorsal horn
TNF-α: Tumor necrosis factor-α
TrkB: Tyrosine-protein kinase B
